# TET2 cascade: a novel regulator of chromatin structure and leukaemogenesis

**DOI:** 10.1038/s41392-024-02101-x

**Published:** 2025-01-06

**Authors:** Wolfram C. M. Dempke, Klaus Fenchel

**Affiliations:** 1 Medical Clinic III, University Medical School, Munich, Germany; 2 Department of Oncology, Cancer Centre, Saalfeld, Germany

**Keywords:** Oncology, Stem cells

In the most recently published research article in *Nature*,^1^ it has been demonstrated for the first time that the TET2 regulates the chromatin structure and leukaemogenesis in stem cells and leukaemia cells via MBD6 (binds 5-methycytosine residues in RNA) and NSUN2 (a RNA methylase). This important finding might pave the way for the development of highly specific novel therapeutic approaches for TET2-mutated cancers.

Acute leukaemias represent a biologically complex, molecularly and clinically hetero-geneous group of haematologic malignancies which are characterized by multiple somatically acquired mutations that affect genes of different functional categories. Mutations in genes encoding epigenetic modifiers, such as DNMT3A (DNA Methyltransferase 3α), ASXL1 (Additional Sex Comb-like 1), TET2 (Ten-Eleven Translocation 2) and IDH1,2 (Isocitrate Dehydrogenase 1,2) are commonly acquired early during the leukaemogenesis process and are known to be present in the initial leukaemia clone.

Methylcytosine dioxygenases (encoded by TET1–3) are known to be critical enzymes for the oxidation of 5-methylcytosine residues within the RNA to generate 5-hydroxy-methylcytosine, and thereby play a key role for the regulation of gene expression in leukaemias which then drives leukaemogenesis and progression of malignant hematopoietic stem cells (HSCs).^2^ Albeit TET2 is considered to belong to the group of tumour-suppressor genes, mutated TET2 (only TET2 has been found to be altered in leukaemias) is known to be a key driver for leukaemogenesis. Interestingly, TET2 has been found to interact with other genes such as FLT-3 (FMS-like Tyrosine Kinase 3) and WT1 (Wilms Tumour 1). In this regard, Maifrede et al.^3^ have provided the first evidence that decreased DNA repair mechanisms are associated with the disrupted binding of mutated TET2 with WT1 suggesting that the finding contributes to the high degree of chemotherapy resistance and poor prognosis of patients with acute leukaemias harbouring TET2 mutations.^2,3^

In the recent years, several lines of experimental evidence have demonstrated that TET2 mutations can be detected in a significant number (12–34%) of myeloid malignancies and lead to DNA hypomethylation^1^ and subsequently to an open chromatin reading frame (increased chromatin accessibility associated with TET2 mutations), which is known to be a critical driver for malignant HSC self-renewal and carcinogenesis.^2^

**Fig. 1:**
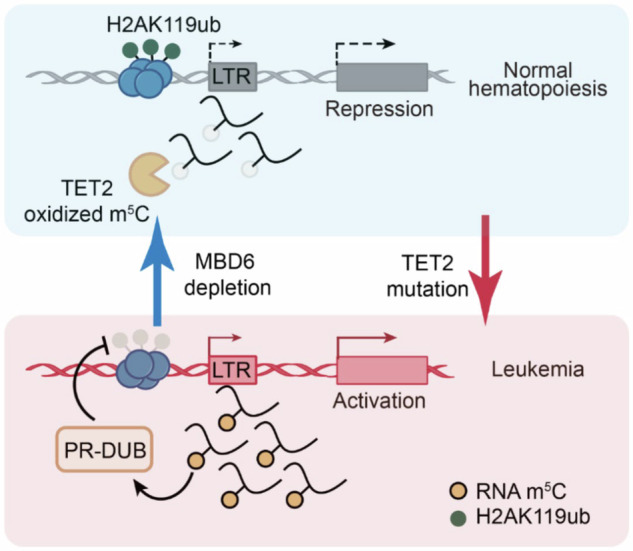
Proposed model of TET2 and MBD6 pathways in normal haematopoiesis (blue) and in leukaemogenesis (pink). During normal haematopoiesis, non-mutated TET2 triggers oxidation of m^5^C, which results in repression of tumour promoter genes (inhibition of the MBD6-mediated de-ubiquitination of H2AK119ub). However, if TET2 is mutated (TET2 deficiency), MBD6 is no longer antagonized, and H2AK119ub is erased by PR-DUB complexes resulting in an open chromatin reading frame with subsequent activation of gene expression profiles. LTR long-terminal repeat, PR-DUB polycomb repressive de-ubiquitylase, H2AK119ub histone 2A (with monoubiquitinated lysine-119), m^5^C retrotransposon RNA 5-methylcytosine (figure adapted from ref. ^1^)

In addition, TET2 has also been shown to antagonize the methyl-CpC-binding protein MBD6, which is involved the de-ubiquitination process of the histone protein H2A. However, if TET2 is mutated, MBD6 is no longer inhibited and, in turn, recognizes retrotransposon RNA 5-methylcytosine (m^5^C) residues, which then will lead to the recruitment of the BAP1 (BRCA1-associated protein 1) complex including PR-DUB (polycomb repressive de-ubiquitylase) with subsequent H2AK119ub deubiquitinating. This results in significantly reduced intracellular H2A levels and subsequently leads to increased open chromatin and transcription of genes maintaining the malignant phenotype^2^ (Fig. 1).

Methylation of RNA molecules is the most common post-transcriptional modification that allows the cellular machinery to control various biological functions. However, the underlying molecular mechanisms are only poorly understood. One of the most critical methylations of RNA molecules is m^5^C at position 34, which is the key driver for cellular proliferation, differentiation, and apoptosis. The regulatory process of methylation and de-methylation is facilitated by “writers” (responsible for introducing m^5^C into the RNA) such as DNA methyltransferase 2 (DNMT2) and NOP/Sun domain family member 2 (NSUN2) (RNA methyltransferase), and “erasers” (removal of m^5^C by oxidizing it to 5-hydroxymethyl-cytosine) such as TET2 and α-ketoglutarate-dependent dioxygenase (ABH1) (reviewed by ref. ^4^).

RNA retrotransposons (class I transposable elements) are molecules that can be integrated into the host DNA by converting their transcribed RNA into a cDNA by the reverse transcriptase (found in 45% of the human genome). These retrotransposons are known to be critical for the chromatin structure and its maintenance. However, several lines of research have provided compelling evidence that retrotransposons are repressed by both DNA methylation and histone modifications resulting in epigenetic silencing.^5^

In a remarkable research article published most recently by Zou et al.,^1^ the research group provided the first evidence that the activity of m^5^C-containing RNA can be regulated by TET2 oxidation of m^5^C resulting in a more closed chromatin structure and less HSC proliferation. In line with this observation, knock-down of the “writer” NSUN2 also led to a more closed chromatin structure and depletion of HSC proliferation. Unmutated TET2 was also found to prevent the MBD6 protein from binding to m^5^C-containing RNA molecules. By contrast, mutated TET2 (loss-of-function mutation) was unable to remove the m^5^C residues, and MBD6, a RNA-binding protein, was found to preferentially bind to m^5^C-containing RNA with subsequent deubiquitination of histone 2A (via the BAP1/PR-DUB complex) resulting in an open chromatin reading frame, and thereby triggering leukaemogenesis. In addition, using SKM-1 cells (acute myeloid leukaemia [AML] cell line harbouring a TET2 mutation), the authors could also demonstrate that a MBD6 knock-down resulted in a complete growth inhibition of these AML cells with comparable results seen for NSUN knock-out cells, and similar findings have been found for human hematopoietic stem and progenitor cells. Controversially, in TET2 knock-out human AML cell models (K562) increased histon2AK119ub levels were found, and TET2 deletion led to activation of genes thought to be responsible for the leukaemogenesis initiation.

Overall, this experimental study demonstrated for the first time that the NSUN2-TET2-MBD6-BAP1/PR-DUB axis in human leukaemias (and most likely in other malignancies as well) is the key underlying mechanism for leukaemogenesis and carcinogenesis in TET2-depleted cells. The outstanding research of He and co-workers^1^ provided the first evidence that mutated TET2 and its downstream protein MBD6 regulate the chromatin structure in leukaemias which will pave the way for the development of highly specific novel therapeutic approaches for TET2-mutated haematological cancers in the near future.
